# Let There Be Light: Photo-Induced Reaction Synthesis—Advancing Manufacturing Horizons

**DOI:** 10.3390/ma19163439

**Published:** 2026-08-13

**Authors:** Shanae Brachtl, Rene Rodriguez, Kiyo Fujimoto

**Affiliations:** 1 Department of Chemistry, Idaho State University, 921 S 8th Ave, Pocatello, ID 83209, USA; vanlshan@isu.edu; 2 Idaho National Laboratory, 1955 Fremont Ave, Idaho Falls, ID 83415, USA; kiyo.fujimoto@inl.gov

**Keywords:** photo-induced reaction synthesis, metal synthesis, ceramic synthesis, semiconductors, lasers, additive manufacturing, feedstock limitations

## Abstract

Photo-induced reactions, also known as photochemical reactions, are chemical processes that are initiated or driven by the absorption of light energy. Photo-induced reactions have been applied in a variety of fields from organic synthesis to polymer curation. One such application of much current interest is additive manufacturing (AM)—a technique used within a variety of fields to produce intricately shaped components. Currently, additive manufacturing is utilized within the solar, medicine, electronic, and nuclear application industries, making it a diverse and expansive system of production. Present reviewed AM production methods include laser powder bed fusion (L-PBF), stereolithography (SLA), direct energy deposition (DED), and selective laser sintering (SLS). However, the literature points to the need for expansion of feedstock capabilities as the horizon broadens to extreme environments like nuclear reactors, a solution which might be found in photolytic reaction synthesis. This review examines photo-induced synthesis most applicable to additive manufacturing, focusing on four key reaction types: oxidation–reduction, combustion, decomposition and polymerization. Past and present applications, future challenges, and emerging opportunities regarding photolytic reaction synthesis are analyzed. This assessment provides insights into the current state and future potential of these reactions in advancing additive manufacturing technologies.

## 1. Introduction

### 1.1. Principles of Photo-Induced Reaction Synthesis

Photo-induced reactions, also known as photochemical reactions, are chemical processes that are initiated or driven by the absorption of light energy. Photothermal, photochemical, and plasma-mediated processes represent three fundamental pathways through which photon-driven reactions occur [1]. Photothermal processes arise when absorbed light is converted into localized heat, enabling temperature-dependent transformations such as decomposition, sintering, melting or phase change within targeted regions of a material [2]. Photochemical processes rely on photon-induced electronic excitations that directly trigger chemical reactions, including bond cleavage, reduction, oxidation, ligand removal, or polymerization, often at significantly lower temperatures than thermally driven routes [3,4]. Plasma-mediated processes occur when high-intensity or ultrafast irradiation generates a transient ionized environment at the material surface or within the surrounding medium. The plasma facilitates rapid bond breaking, surface activation, densification, or reactive species formation [5,6]. A summary of photo-driven mechanisms is presented in Table 1.

Although light is often considered to be electromagnetic radiation (EM) in just the visible range, this review will include a few examples outside the visible range. This review will include lasers as a major source of electromagnetic radiation, as they amplify light via stimulated emission of radiation, thereby producing a highly concentrated emission of energy with unique properties, including coherence, monochromaticity, and collimation (Table 2).

Lasers have evolved significantly over the years since the first laser became a reality in the early 1960s [7,8]. Both continuous wave (CW) and pulsed lasers deliver significantly more power in a smaller footprint than was even possible 15 years ago, reflecting sustained progress in high-power architectures and efficiency across solid-state and fiber formats [9,10]. Large water-cooled ion lasers, such as argon (Ar) and krypton (Kr)-ion lasers, have been replaced by more efficient and compact alternatives like diode lasers and diode pumped solid-state (DPSS) lasers, where the solid-state material (e.g., Nd:YAG crystal) serves as a gain medium for amplifying light [11]. These DPSS systems also include high-power fiber lasers (where the optical fiber itself is the gain medium), can now generate beams on the order of kilowatts to tens of kilowatts in continuous mode [9,10,12]. Pulsed laser technology has evolved significantly in the same time period, as pulse-widths and repetition rates have evolved from the nanosecond to microsecond realm of flashlamp-pumped dye-laser, solid-state lasers, and excimer lasers with repetition rates in the tens of Hertz to today’s picosecond and femtosecond ultrafast lasers with high repetition rates in the kHz to MHz range [6]. Pulsed lasers can achieve high pulse powers in the range of hundreds of kilowatts to gigawatts due to their short pulse-widths and energy storage [7]. Such capabilities have opened new frontiers in materials processing from precision drilling, cutting, welding and engraving to a host of photo-induced synthesis techniques where focused light triggers chemical or phase transformations in materials [13]. DPSS lasers have concentrated, high-power output capability and are used in the industry for drilling, cutting, welding, ablating, and engraving [14,15].

Photolytic reaction syntheses induced by lasers have been used in diverse areas including organic material synthesis and ceramic production. This paper examines the synthesis of transition metals (like copper), graphene, ceramics, polymers, and semiconductors. Current reactions employing photo-induced synthesis include a range of processes, such as oxidation–reduction, combustion, decomposition, and polymerization. Notably, the applications of photo-induced reactions extended into the field of additive manufacturing, highlighting their significance in modern fabrication techniques.

Within the field of additive manufacturing (AM), photon-driven processes have been heavily utilized primarily for shaping materials through photothermal mechanisms rather than altering their chemistry to build parts to cure polymers or melt metal powders to solidify or fuse feedstock layer by layer rather than fundamentally changing their chemistry. Traditionally, AM builds objects layer by layer from feedstock materials (powders, filaments, resins, etc.) without altering their base chemistry. This process typically begins with a computer-aided design (CAD) file to generate a 3D model of the product which is then generated into a file type comprising geometric shapes, often a standard tessellation language or standard triangle language (STL) file [16]. The file is then sliced into thin increments to be deposited layer by layer. Each method of printing will vary after the STL file is formed.

Printing methods that utilize photon-driven processes include laser powder bed fusion (L-PBF), stereolithography (SLA), Digital Light Processing (DLP), direct energy deposition (DED), and selective laser sintering (SLS). Laser powder bed and its polymer counterpart SLS uses scanned laser spots to melt or sinter regions of a powder bed, bonding particles into a solid layer that matches the cross-section of the digital design [17]. Over repeated processes of spreading powder and laser-scanning, an intricate 3D part is fabricated. In DED, a laser, often a high power CO_2_ or fiber laser, is focused co-axially with a stream of metal powder or wire feedstock; as the laser moves, it melts incoming material, enabling the fabrication of large metal parts with near-net shape [18].

For polymeric materials, vat photopolymerization methods like stereolithography (SLA) relies on a UV laser (often a 355 nm or 405 nm UV laser) to initiate an exothermic polymerization reaction to cure the resin which creates the layers of deposition to print thermosets and cross-linked elastomers, and the entire SLA process and system has been thoroughly reviewed by Huang, Qin, and Wang previously [19]. A related approach, DLP, uses a digital micromirror array to project entire image slices to cure photosensitive resin layer by layer with a UV light flash instead of a scanning laser [20]. SLA and DLP AM resins generally rely on acrylate-based free-radical chain-growth polymerizations [20].

Among other laser-enabled AM techniques, two-photon processing (2PP) stands out for its ability to build components with sub-micron precision by harnessing nonlinear optical absorption [21]. First described theoretically by Maria Göpert-Mayer in 1931 In 2PP, an ultrafast, high-intensity laser (typically a mode-locked Ti:sapphire emitting 800 nm pulses of <100–200 fs duration) is tightly focused into a photoresponsive material [22,23]. Two photons are absorbed near simultaneously within a femtoliter-scale voxel, which supplies the energy equivalent of a single higher energy photon. Photopolymerization occurs only at the focal point because of this localized two-photon exciting. Commercial 2PP resins are commonly acrylate or epoxy-based formulations similar to those used for SLA/DLP, but with photo-initiators optimized for two-photon absorption. This technique has demonstrated a broad materials palette for AM that ranges from cross-linked polymer microstructures to photoreduction of noble metals within a resin [24,25,26,27,28,29]. 2PP also supports templated pathways to inorganic materials. Hybrid organic–inorganic and pre-ceramic resins have been printed and then globally converted to oxide and ceramic microstructures while maintaining complex 3D geometries [30,31,32,33,34]. While scale and throughput remain limited, 2PP remains one of the few AM methods to successfully show in situ synthesis from precursors alongside high fidelity 3D fabrication.

Direct-write deposition technologies (ink jet printing, aerosol jet printing, micro-extrusion methods) enable maskless, digitally controlled patterning of materials by depositing functional inks or pastes where needed. These processes traditionally require separate post-deposition steps to achieve final properties, and are used for thin-film development for a wide range for industries including biomedical, space and nuclear, among others [35,36,37,38,39]. Similar to 2PP, these techniques have demonstrated a large material palette that includes 2D materials, polymers, metals, semiconductors and ceramics [40,41,42,43,44,45,46,47,48]. Recent advances combine focused light sources with these direct-write printers to induce in situ material transformations to cure, sinter or even drive chemical reactions during the printing process rather than after [49,50,51].

These laser- or light-driven AM techniques have revolutionized manufacturing by allowing the creation of complex geometries from metals, plastics and ceramics. However, the photonic energy in these conventional AM processes is typically used to induce phase changes (melting, solidification, or cross-linking) rather than to effect any change in chemical composition. As a result, the materials microstructure and phase content are determined by the feedstock composition and thermal history, not by synthesizing materials during printing.

Additive manufacturing of metals and ceramics has advanced rapidly, but current techniques face fundamental material challenges that limit part quality and material selection. The range of compatible feedstock materials is constrained, as only certain alloys and polymers can be readily prepared in the needed form (powder, wire, or resin) with the required purity, flow characteristics, and shelf stability. Highly reactive materials like titanium or aluminum must be handled as powders in inert conditions due to oxidation and combustion risks, and their powders can degrade or become contaminated with repeated reuse [52]. Additionally, for parts produced from techniques such as L-PBF, printed metal components often exhibit high surface roughness and microstructural inhomogeneities, necessitating time-consuming post-process refinements to meet conventional manufacturing standards [53]. These constraints, ranging from feedstock limitations (material availability, shelf-life, handling, novel material development, etc.) to extensive post-print thermal or mechanical treatments motivate the search for new approaches that can overcome the efficiency and material shortcomings of traditional AM workflows.

An emerging class of 3D printing techniques harnesses photo-induced in situ chemical reactions during printing as a true synthesis pathway for advanced materials. Embedding in situ chemical reactions into AM imparts enhanced functionality to printed parts by making it possible to create new phases or refine microstructures on the fly. In these processes, a laser beam, UV/visible light or intense pulsed lamp triggers transformations of material precursors into functional solids as each layer or feature is deposited. This approach goes beyond conventional laser melting or simple UV curing, instead harnessing photon energy to induce photochemical reductions, decompositions, or phase transformations that yield metals, ceramics, or other compounds from feedstocks.

Photochemical processes inherently compatible with additive manufacturing’s localized framework make it feasible to drive chemical transformations exactly where and when they are needed during part fabrication. Integrating such photon-activated in situ synthesis within AM could vastly expand the materials library and microstructural tuning achievable in printed components. Photon-driven chemistry has the potential to elevate 3D printing from a geometry-centric technique to a versatile materials manufacturing platform. This review will spotlight its promise as a catalyst for the next generation of advanced manufacturing.

### 1.2. Advantages of Photo-Induced Reaction Synthesis

The use of laser-driven photo-induced synthesis has significant implications for additive manufacturing processes, and the advantages of photo-induced reaction synthesis begin with efficiency of production. First, it enables a one-step fabrication of functional materials. Compared to typical chemical workups, photo-induced chemistry does not rely on harsh or aggressive solvents for synthesis and can often be carried out at room temperature, significantly reducing waste and making the process more environmentally friendly [54]. Moreover, the use of lasers in photo-induced reactions allow for precise spatial and temporal control over the reaction, which enables rapid and clean processing while also allowing for localized and highly controlled reactions that are not easily achievable with other light sources. The ability to focus light and laser light energy with pinpoint accuracy makes it possible to manipulate material properties at micro- and nanoscale levels. This is especially advantageous for the fabrication of intricate structures, and when combined with an AM process, it enables the fabrication of complex, customized parts with exceptional material properties.

Additionally, utilizing a laser-based approach for photo-induced reactions facilitates materials synthesis in non-equilibrium conditions, leading to unique microstructures and providing pathways for selective reduction and novel material synthesis. The tunable energy input and compatibility with additive manufacturing further enhance the versatility of laser-driven processes, allowing for scalability, high-throughput combinatorial synthesis, and the production of advanced materials with tailored properties.

### 1.3. Disadvantages of Photo-Induced Reaction Synthesis

In some cases, excitation of the reactants may lead to several different reaction pathways reducing the yield of the product of interest. Photochemical side reactions, fast back electron transfer processes and recombination reactions, may considerably limit the efficiency of photocatalytic reactions, and the photochemical decomposition of the catalyst may further lead to the fast termination of photocatalytic cycles [55]. Mechanisms associated with photo-induced reactions are often difficult to discern and can be complicated. This exacerbates attempts to minimize unwanted side reactions. If the irradiation is non-uniform or if the light is outside the correct frequency range, then the reaction efficiency is suboptimal. Additionally, these processes demand very high light intensities or doses, which makes it so that equipment needed to produce laser excitation with the correct frequency and power can be specialized and costly, although laser technology has advanced significantly in the past few years.

### 1.4. Hazards of Laser Operation

Lasers are high-powered sources of monochromatic light. In addition to the cost associated with their purchase, additional funding is required for beam manipulation (optical components), and also for personal protection equipment. Goggles are required which block laser light in the correct frequency range. (See Table 3 for a summary list of merits and demerits regarding photo-induced reactions).

## 2. Examples of Photo-Induced Reactions

### 2.1. Oxidation–Reduction and Combustion Reactions

One of the current photo-induced reaction synthesis methods applies oxidation–reduction (redox) chemistry to promote synthesis of metals, graphene, and other materials. In the context of AM, the most compelling use for these reactions is to produce or modify materials from chemical precursors during the printing process rather than requiring a separate high temperature furnace or chemical bath.

#### 2.1.1. Transition Metals

The earliest application of direct reduction of a metal’s oxidation state was found in the 1987 Ultramicroscopy study investigating the electron-beam-induced reaction of transition metal oxides to metallic lower oxides [56]. Here, researchers selected transition metal oxides with maximum valence and reduced them to binary oxides with metallic nature through electron irradiation. Although this is not, strictly speaking, a photo-induced reduction, the study was significant as it demonstrated the use of an excitation beam to produce surface oxide layers and opened the door to further possibilities.

Laser ablation has become a widely used method for driving transition metal redox reactions to form both metallic and ceramic materials. This method utilizes both pulsed wave and continuous wave lasers and is adapted to a broad range of physical states of reactants and products [57,58]. Multiple studies have demonstrated that laser ablation can generate reactive metal atoms, clusters and oxides from a wide variety of transition metals to include iron, iron oxide, silver, nickel, platinum and copper, among many others [58,59,60,61,62]. Prior reviews provide extensive detail on the mechanisms, processing conditions, and materials formed through laser-ablation-based synthesis, underscoring its significance in both fundamental and applied research [57,63,64,65]. An illustrative example is the gas phase oxidation of copper, in which researchers used laser ablation to vaporize solid copper to study its oxidation kinetics in controlled environments. In this study, the scientists observed different oxidizing environments (O_2_, N_2_O, and NO_2_) in which CuO was formed through laser ablation and monitored with laser-induced fluorescence [66]. This study used two lasers, XeCl (308 nm) and ArF (193 nm), varied pressures (100–500 mTorr), and several laser fluences (1–6 J/cm^2^) to determine the best environment for maximum CuO production (100–250 mTorr and 6.0 J/cm^2^). The most effective environment was found to be NO_2_, with N_2_O not far behind, and the O_2_ environment was least effective [66]. Significantly, these studies show the versatility of photo-induced reactions with the various physical states of reactants and products.

In a second study focusing on copper, the fabrication of a Cu electrode via laser-induced direct reduction was explored to develop an environmentally friendly and quick method of manufacture. Synthesis involved the suspension of copper oxide nanoparticle powder within a liquid reducing agent (ethylene glycol), which was then deposited onto a glass substrate. From there, a 0.4 W ytterbium-doped fiber laser was used to develop optimized parameters to successfully convert CuO to Cu film in a direct reduction [67]. This method utilizes the stability and cost-effectiveness of copper oxide to produce the more expensive copper electrode with a specific electrical resistance of 31 µΩ × cm with the assistance of a laser-induced reduction reaction [67]. The specific resistance of pure copper at 20 °C is 1.7 µΩ.

In a third study involving photo-assisted redox of copper, again looking at cost-effectiveness, investigators took advantage of laser-induced photochemical conversion of metal complexes to metals in CuSO_4_**^−^** and CuCl_2_**^−^** electrolyte solutions [68]. The copper complex was reduced by passing an Ar^+^ multi-wave laser (5–500 mW) through the desired solution which initiated the conversion to the metal to be deposited. Here, the laser acted as an auto-catalyst on the surface to provide active sites and electrons for the reduction of the complex molecule and initiated reaction.

Unique copper structures can also result from the reduction of Cu_2_O. In one study, the combination of an applied electrochemical bias and illumination was used to mediate the deposition of copper metal on the [1,0,0] facets of Cu_2_O while the interior is [1,1,1] Cu_2_O, resulting in a shell-like structure. In this experiment, the illumination source was a broadband, ELH-type light bulb and the electrochemical apparatus was a BioLogic VSP-300 potentiostat/galvanostat. Photoelectrochemical experiments were performed using gold deposited on glass for the working electrode, platinum gauze-wire as the counter electrode, and Ag-AgCl as the reference electrode. The cuprous oxide microcrystals were electrodeposited from an aqueous 0.02 M Cu(NO_3_)_2_ solution. This study revealed that the combination of an applied electrochemical bias and illumination can control the facet-dependence of photochemical reactions on the surface of Cu_2_O, which complemented other studies where photo-excitation led to the deposition of metals and unique morphologies that can be grown using light in combination with a potential.

Other reactions involving transition metals, where photo-excitation facilitated their deposition, include Ag, Au, and Ag-Au alloy [69,70]. In these instances, conversion “is assisted by the generation of mobile charge carriers that can be extracted from the material to oxidize or reduce surface-adsorbed species. The extraction of photoexcited charges can also be used to grow or transform the material itself” [71].

Patterned deposition of transition metals represents another area of interest with potential applications in additive manufacturing processing. In one study, direct deposition of metal nano-patterned structures was achieved through laser-induced reduction of metal ions on a graphene film with feature sizes as small as ~300 nm with 532 nm light. Deposition of several metals on the graphene film was achieved and included Au, Ag, Pd, Pb and Pt metals [72].

#### 2.1.2. Graphene

Another promising material synthesized by photo-induced reactions is graphene. Researchers have investigated cost-effective and environmentally friendly approaches. In a chemical-free approach, graphene is rapidly synthesized with the help of a focused laser-induced self-propagating reaction. The reduction reaction began with graphite oxide flakes in a quartz tube and a continuous wave semiconductor laser (808 nm) with laser power of 1 W and power density of 127 W cm^−2^ focused to a small spot size around 1 mm^2^ to trigger the self-propagating reaction process to produce graphene layers [73]. Further interest in graphene has led to the laser-induced reduction of graphene oxide powders with an ultraviolet pulsed laser. Optimized parameters such as repetition frequency of 100 kHz, galvanometer scanning speed of 50 mm/s, 10 irradiated cycles, and laser fluence of 0.438 mJ/cm^2^ were used to develop reduced graphene oxide with an oxygen content of 34.94% compared to the initial oxygen content of 81.15% [74]. With the intent to form graphene films with high productivity, researchers took photo-induced reactions a step further by introducing a cylindrical lens to produce a wider laser beam. In a subsequent investigation, a 40 to 60 W, 10.6 µm, laser beam, from a Cortyard conventional CO_2_ laser engraver, was expanded with a lens under a nitrogen environment to reduce graphene oxide films for use in supercapacitors [75].

In an effort to combine photoreduction and device fabrication, researchers developed a method to utilize laser-induced programmable N doping with simultaneous reduction of graphene oxides under an ammonia environment. In this experiment, a femtosecond pulsed laser (800 nm) with 120 fs pulse duration and 80 MHz repetition rate was used and the doping level was controlled by varying the laser power (8–12 mW) [76]. This method provides complex control to provide micropatterns while the simultaneous formation avoids the heating step used in diffusion, saving time and energy while being precise [76].

#### 2.1.3. Halides

The use of halide compounds for the deposition elemental substance and semiconductors has also been investigated in photolytic oxidation–reduction processes. By using plasma to activate chemical reactions in a precursor gas, volatile BCl_3_ with H_2_ and CH_4_ led to conversion to the pure elemental isotopes of boron and carbon and isotopic compound of boron carbide [77]. The laser used was a Nd:YAG laser with a wavelength of 1064 nm, a 15 ns duration, 800 mJ pulse energy, and 5 Hz repetition rate.

Laser-induced dielectric breakdown (LIB) of a metal in the presence of reactive gases is another oxidative method used for synthesis and deposition of materials for use in technology and industrial applications. In one study, LIB of molybdenum, in the presence of H_2_ and BF_3_ gases, at pressures of 30 and 160 Torr, produced nano-powder of molybdenum boride with a characteristic grain size of 100 nm, and at pressures above 160 Torr, Mo nano-powder with a grain size < 30 nm was obtained. For this study, a pulsed Nd:YAG laser (1064 nm) with a pulse energy of 800 mJ, pulse duration of 15 ns, and repetition rate of 5 Hz was used [78].

#### 2.1.4. Ceramics

Beyond metal redox chemistries, laser ablation methods have also been utilized to synthesize ceramics [79]. Newer approaches for ceramic material synthesis involve reactions catalyzed by the assistance of photons, in the form of microwaves. One important type of redox reaction is self-propagating high-temperature synthesis (SHS), which is used for the synthesis of high temperature composite and metastable phase materials. Conventionally, the SHS process occurs on the surface of the material by heating with a laser, a heating coil or in a furnace, and the surface always reaches a higher temperature than the center.

In one study, using the model reaction of the production of aluminum oxide and titanium carbide by the reaction 3TiO_2_ + 3C+ 4A1 → 3TiC + 2A1_2_O_3_, a different approach using focused microwaves was employed to provide heating from the center to the surface. The authors made use of the idea that microwaves heat non-conductive compounds more uniformly in a volumetric way, and the combustion occurs from the center outward to the surface. The newer approach led to a different morphology and provided for a more complete consumption of the reactants. The microwave-induced and conventionally heated SHS reactions both synthesized similar looking ceramic composites, but the microwave SHS samples had both higher density and more uniform microstructure. The whiskers of product material were smaller and contained solid cores with microwave heating, as compared to the conventional case, where the product whiskers were hollow and a bit longer. Microwave heating also proved to be energetically more efficient and therefore was offered as an energy efficient alternative to synthesize ceramics [80].

The microwave-assisted SHS process has been employed in the production of other ceramics as well. Alumina-silicon carbide ceramics were produced from silicon dioxide, aluminum, and carbon powders. The concentration of a potassium nitrate as an initiator was varied to determine its effect on the product, and the reaction was performed in a microwave operating in a multimode fashion at a frequency of 2.45 GHz [81].

### 2.2. Decomposition Reactions

Decomposition reactions have also been investigated through the lens of photo-induced reactions. In one study, a CO_2_ laser operating at approximately 300–400 mJ/pulse at the P-22 line near 10 microns was used to induce decomposition of carbon and silicon containing gases within a flow reactor to produce carbon and silicon cluster beams of ultrapure nanoparticles. This method utilizes gas-phase reactants for ease and efficiency. A mixture of C_2_H_6_ and SF_6_ gases in an argon buffer gas were used for production carbon clusters whereas SiH_4_ gas in a helium buffer gas was used for silicon clusters, and the gases flowed in a perpendicular motion from the focused laser radiation. Strong luminescence was formed at the reaction center where the laser was tuned to near maximum absorption of the gases; soot was collected and clusters were extracted and passed through the mass spectrometer [82]. The decomposition products were then introduced into a molecular beam source chamber for ionization of the cluster and determination of the cluster size by time-of-flight mass spectrometry. Either a doubled Nd:YAG laser (532 nm) or an excimer laser (193 nm) at an energy of ~10–50 mJ/pulse were used to ionize the clusters.

### 2.3. Polymerization Reactions

A common application for photo-induced reactions is in the production of ceramic polymers by the polymer-derived ceramic (PDC) route. The PDC route uses monomers or polymers with wet chemical techniques to fabricate a pre-ceramic precursor, the polymerization of which leads to the desired final ceramic product after heating the pre-ceramic precursors to a high temperature. One particular study of the polymerization of Si/B/C/N random covalent network ceramics employed a photo-assisted PDC route [83]. These Si/B/C/N network ceramics often have high thermal, chemical, and mechanical stability in oxygen free atmospheres, even at temperatures up to 2000–2200 °C. The authors compared the Si/B/C/N polymer produced by the photo-induced PCD method to the Si/B/C/N polymer produced by more conventional methods. Using a single source precursor and UV-induced polymerization, a pre-ceramic polymer of B, B’, B”–(dimethyl)ethyl-acrylate-silyloxyethyl-borazine was synthesized [83]. After dropwise addition steps with various temperature controls and liquid reactants, a photo-initiator was added and ultraviolet light irradiation was used to produce a-CSEB polymer at 92% yield. Subsequent treatment of this polymer by heating it at a high temperature of 1100 °C for 4 h under a nitrogen atmosphere was used to obtain black ceramic. The material was characterized by nuclear magnetic resonance (NMR) and Fourier transform infrared (FTIR), showing evidence of six-membered borazine ring units, B_3_N_3_, while the polymerization properties were analyzed with Photo-DDSC with clear exothermic phenomena. Researchers found efficient UV-curing properties shown with exposure as low as 40 s where the double-bond conversion rate was approximately 92.75% [83].

Polymer-based resin materials are an important material in AM. One problem encountered in their use is the incomplete association between the layers of the deposited materials. This lack of layer-to-layer association appears in SLA and DLP AM processes. Cross-linking the molecular chains between layers is purported to improve the interlayer association and the overall final product made with the AM technique [84]. A recent study touted a new type of photo-reversible cross-linking resin material that can be cured for cross-linking with 354 nm light and cleaved with 254 nm light [85]. The new resin consists of a combination of polyvinyl alcohol and coumarin linked together by an esterification process. Measurements of the adhesion properties, the thermal conductivity, and the oxygen barrier performance of materials produced with this resin suggest better adhesion, conductivity and barrier performance when compared with materials made with a PVA resin. The ability to irradiate the resin with 254 nm UV light to cleave suggests that the resin will be more recyclable.

### 2.4. Semiconductors

Semiconductors are an important class of materials with applications in many areas. For example, they are useful as catalysts, as photon detectors, and as electronic components in computers and other electronic devices. Light can be employed in both their production as well as in their patterning and may also enhance their use as catalysts. In one example, the catalytic activity of MnO_x_ films for the oxygen evolution reaction (OER) was significantly enhanced during synthesis using a multipotential deposition under illumination with a 405 nm light-emitting diode. The illumination provided for a beneficial tuning of their composition and morphology during the MnO_x_ film growth, and the MnOx films synthesized under illumination provided for a significantly higher OER activity [86]. For MnO_x_ films, the presence of Mn^3+^ ions in the MnO_x_ film can greatly enhance its catalytic activity, and manganese oxide films synthesized by multipotential electrodeposition produce MnO_x_ films with a higher Mn^3+^ ratio than films synthesized by other electrochemical methods. Also, it is known that Mn^3+^ ions in tetrahedral sites can be kinetically trapped by structural constraints and forced to remain as Mn^3+^. The conclusions of this study were that the light provided for a tuning of the morphological film structures into ones that trapped the Mn^3+^, providing for the persistence of structurally stabilized Mn^3+^ in the manganese oxide films which enhanced its function as a catalyst for the OER.

Light also acts as a colloidal patterning method for semiconductor nanoparticles using light-triggered modulation of their surface charge. In one study, upon irradiation with low optical intensity (~10 mW/cm^2^) UV light for approximately two minutes, ZnO nanoparticles absorbed photons causing surface-bound citrate ligands to be released. Loss of the citrate anions from the ZnO nanoparticles makes them more positive, and they are then attracted to a negatively charged substrate. Zn^2+^ ions are formed during illumination, and they can bind to nearby nanoparticles and produce stacked multilayer ZnO nanoparticle structures. Through appropriate selection of the ligands on the surface, both positive and negative patterning on the same substrate is possible, and overall allows for patterns of ZnO superstructures on a substrate [87,88].

The crystalline phase can also be controlled by laser illumination. Light can also be used to alter and control the crystalline phase of certain semiconductors. In one report of laser-induced localized phase transitions of TiO_2_ thin films, the formation of crystalline microstructures due to laser treatment was observed. Laser irradiation under vacuum results in an anatase-to-rutile phase transition, and irradiating the rutile region in air changes the crystal structure back to anatase, despite the thermodynamic stability of rutile. One possible explanation is that the laser light is absorbed by defects in the material, causing localized excitation of the electrons into a mixture of amorphous and crystalline regions. The phase of the crystalline regions depends strongly on the ambient conditions [89]. Structural changes are also known to occur in amorphous semiconductors. Laser irradiation of Ge_46_S_54_ chalcogenide glass films leads to an alteration the Ge–S backbone leading to a structure depleted in germanium as a result of photo-induced oxidation of some of the available germanium [90].

Semiconductors, as layered 2-dimensional (2D) sheets, have recently drawn significant attention due in large part to the ability to tune their physical properties due to the microscopic interactions between layers [91]. 2D sheets of MoS_2_, graphene, and BeSe_2_ have been studied [92,93,94]. In a recent study, a strong THz electric field perpendicular to the layers was recently shown to be an effective way to control these types of properties [95]. A 2D semiconductor with an electric field applied in an out-of-plane direction can have a shift in its ground state optical transition due to the quantum confined Stark effect (QCSE). Assuming that this electric field in a semiconductor pre-exists, if an additional electric field is applied in the same direction, it can weaken or enhance the Stark effect already present in a positive or negative way based on its polarity. The authors reported that the application of a supplemental electric field in the THz range, caused a blueshift or a redshift of the optical transition energy when the THz field was polarized similarly or oppositely.

Enhanced doping in semiconductors has also been reported when light is applied during the doping. One early explanation is that anion desorbs more easily when photons hit the surface, creating openings in the atomic reconstruction that occurs at the top smooth surface on the crystalline substrate, and this makes it easier for dopant atom incorporation. In one example, in the presence of UV light, Te desorbed more readily from a CdTe surface compared to the amount of Cd that was lost, and this seemingly allowed for incorporation of Sb dopant [96].

To provide a consolidated comparison of the photo-induced synthesis methods discussed in Section 2.1, Section 2.2, Section 2.3 and Section 2.4, Table 4 summarizes each method in terms of reaction type, precursor chemistry, light source, wavelength, synthesized products, key advantages, and relevance to manufacturing.

## 3. Applications

### 3.1. Additive Manufacturing as a New Paradigm in AM

If progress were linear, advances in manufacturing would be limited to a production line. However, with the capabilities of additive manufacturing, the extent of manufacturing has the potential to expand exponentially. Fundamentally, AM enables complexity and customization without proportional increases in cost or time by decoupling the manufacturing complexity from manufacturing difficulty. A conventional production line requires a new tool or setup for each added feature, but a 3D printer can build complex shapes in a single operation. As a result, printing a detailed object is not typically any more labor intensive than printing a simple one. Having voxel-level control means the design space for AM grows combinatorially rather than linearly, as designs with new architectures, internal features, or lattice complexities can be realized with little to no extra overhead. Introducing photo-initiated chemistry into AM expands the potential of its manufacturing capabilities to enable on demand variation in not only shape, but also local material composition and microstructure.

Today, many advanced AM techniques ranging from laser melt powder fusion to vat photopolymerization already rely on highly localized energy in the form of light to build structures rather than changing their chemistry. In these processes, a tightly focused laser or digitally projected image provides energy with micrometer precision, inducing physical or chemical changes only where needed. Crucially, light in AM can serve as more than just a heat source, but also a catalyst for chemistry [97]. One example includes SLA and DLP where UV photons initiate polymerization of liquid monomers, transforming them into solid cross-linked networks in defined regions/patterns. In metal and ceramic AM, light not only melts or fuses powders but can also drive reactions like the photoreduction of metal ions to form nanoparticles or the photothermal decomposition of organometallic and polymer precursors to yield metals, oxides or carbon. At small scales, AM has already proven the power of photon-driven transformations.

Extending this principle, next-generation systems will use photons not just to shape materials, but to drive on-demand chemical transformations during fabrication so the photon itself becomes a digital reagent, triggering reductions, decompositions, polymerizations and phase changes in situ for photon-driven reactive printing. Since these photon-mediated reactions can be executed in different regions at different times simply by steering a beam or projecting a pattern, they offer an unprecedented level of both spatial and temporal control over material synthesis in tandem with shape formation. The main motivation for direct approaches is to eliminate intermediate binders or polymer matrices that would later need debinding and high temperature sintering, which are steps that typically cause significant shrinkage (15–30%) and can introduce cracks or distortion in ceramic parts [32]. This paradigm shift would significantly expand the material library and microstructures accessible via AM to transform 3D printers into flexible chemical reactors that can produce parts with tailored composition and functionality at each voxel.

Merging programmable photochemistry with 3D printing enables in situ material synthesis of materials previously incompatible with additive methods. For example, printing ceramics or functional alloys traditionally requires specially formulated powders or binders for each material, and often extensive post-processing to achieve full density or the desired phase. In contrast, a photochemical AM approach could start from a stable precursor (like a metal salt solution, organometallic, or polymer composite) and use a precise light stimulus to convert it into the target material during the build. This vision has been partially outlined by Yee and Greer who suggested that the future of vat photopolymerization can be reimagined as “3D chemical reactors” in which in situ materials synthesis techniques are employed to widen the scope of printable materials [98].

Integrating material synthesis into the printing process is still in its nascent stages, but steady advances, as detailed in subsequent sections of this review, are bringing it closer to reality.

#### 3.1.1. Additive Manufacturing of Ceramics

Laser-based powder bed fusion of ceramics has attracted growing interest as a pathway to produce complex ceramic parts without binders and multi-step sintering treatments that are traditionally required in conventional ceramic processing. In other words, this enables bypassing debinding and post-sintering process by consolidating ceramic powder in situ to a near final object and would likely minimize issues like shrinkage and cracking [99]. While photochemical and plasma-mediated mechanisms are not widely reported for AM of ceramics, studies have shown that direct conversion of ceramic powder to fabricated parts is achievable. For example, direct powder bed selective laser additive manufacturing has been used to manufacture silicon carbide (SiC) ceramics. Using the direct method, researchers were able to bypass debinding and post-sintering steps while avoiding crack formation, shrinkage, and slumping using a fiber laser (1060 nm) with 300 W maximum power (spot size = 70 μm), laser power of 30–50 W, scanning speed (v) of 50–500 mm/s, and hatching distance of 8.75–87.5 μm [100]. By optimizing laser power (45 W), scan speed (250 mm/s), layer thickness (30 μm), and hatch spacing (35 μm) combined with a hexagonal scan pattern rotated 90° between layers to distribute thermal stresses, they produced crack-free SiC parts of complex geometry at around 81% relative density. Additionally, the printed SiC had a fine microstructure and retained the intrinsic phases of the starting powder except for the small fraction of decomposed material. Notably, these specimens were fabricated binder-free, directly from SiC powder with full density compaction of each layer, demonstrating the feasibility of single step fabrication of SiC. However, the parts showed 5 wt% residual free silicon and about 1 wt% carbon, indicating that the high temperatures reached by laser heating (>2000 °C) caused some SiC to decompose into silicon and carbon during processing. This highlights a general challenge for laser-fusing ceramics, as many technical ceramics do not have stable, congruent melting points at ambient temperature resulting in dissociation or sublimation at extreme temperatures. This introduces difficulties in fully melting materials without thermal decomposition or phase change. As a result, *direct* laser sintering of such materials often yields only partial densification and can leave residual secondary phases from decomposition. In Montón’s case, the residual silicon and carbon were relics of SiC that thermally dissociated. Employing a post-fabrication step such as transient liquid phase sintering or spark plasma sintering could eliminate these residuals and further densify the part [101]. Regardless, this demonstration exemplifies both the promise and hurdles associated with a single step or direct approach.

Reported laser-sintered densities for alumina have reached 85–90% in the best cases, with Abdelmoula et al. (2022) obtaining ~88% relative density by using an “island” scanning strategy and a graphite-doped powder to improve laser absorption, while simple linear and concentric scanning resulted in 75% and 67% density, respectively [102]. Similarly, Liu et al. introduced a dual laser approach for yttria-stabilized zirconia (YSZ) in which a defocused Nd:YAG laser first preheated the powder bed, and reducing thermal shock, before a 1 µm fiber laser scanned each layer. This yielded crack-free 8 mol% YSZ parts at 90–91% relative density [103]. 

#### 3.1.2. Additive Manufacturing of Metals

Additive manufacturing of metals spans from thin-film development to fabrication of large structural components, and is a widely used method of production with various techniques. Well-known methods include laser beam melting of metals, electron beam melting of metals, and laser metal deposition. Each of these techniques is well-established in the industry for high-performance metal components, yet each also faces challenges such as high equipment costs (lasers, vacuum systems, and/or inert gas handling), significant thermal stresses and distortion of parts and limitations in processing materials with very high melting points or lower laser absorptivity [104]. Additionally, direct-write methods have demonstrated a broad range metal printing capabilities through the development of printed sensors and electronics [39,46,47,50]. However, in situ photo-induced processes are a relatively recent and still emerging approach with comparatively few demonstrated routes directly during printing.

To expand the energy sources available for metal AM, researchers have begun exploring photon and electromagnetic approaches beyond the traditional lasers and electron beams. A relatively new and novel method of depositing metal during an additive manufacturing process involves the use of focused microwaves. In the past, microwaves were mostly applied to sinter ceramics or heat metals in bulk, often requiring either susceptors or special conditions because bulk metals reflect microwave radiation [105,106]. Nevertheless, pioneering experiments in the 1990s showed that fine metallic powders can be heated and even fully sintered by microwaves if embedded in *lossy* dielectrically absorbing medium or hybrid heating setup, which helps overcome the reflectivity of bulk metals [107]. Building on this concept, researchers have proposed using localized microwave heating for additive manufacturing with the idea that by concentrating microwaves on a small powder volume would melt or consolidate a layer of metal powder similar to using a laser or electron beam.

A key advantage of microwave-based heating lies in its strongly selective volumetric coupling, as electromagnetic energy can be directed to couple preferentially with the metal or added susceptor and not with the surrounding environment [108]. This minimizes thermal losses and enables AM on temperature-sensitive substrates [109]. Microwaves have been used for joining metal parts together and for the production of metal objects [110,111,112]. Incremental solidification of small quantities of metal powder is an important and basic process in various additive manufacturing processes and its feasibility was perhaps first demonstrated by Shelef and Jerby in 2020 [113]. They introduced a localized microwave heating process which they found to be suitable for bronze and iron-based powders. To yield a 20 mm long 2 mm diameter bronze rod in a sequence of nine steps, an incident power of 180 watts (with ~35 watts reflected) for a duration of 8 s was used. For the iron-based powders, an external static magnetic field was used to help hold the powder in place. By demonstrating all solid-state microwave generation and careful control of the field, the experiment by Shelef and Jerby established the feasibility of microwave-driven AM and suggested that scaling to more complex geometries might be possible with higher power or multiple applicators scanning across a powder bed.

Following this work, researchers advanced microwave AM toward greater precision and functionality. Shoshani et al. (2023) demonstrated a scanning microwave powder bed fusion process [109]. In their setup, a 250 W solid-state microwave source was directed through a split waveguide application with a scanning head, achieving a lateral solidification rate of ~1 mm^3^/s in bronze powders. The density and Vickers hardness of the microwave sintered bronze were comparable to laser-printed bronze, indicating effective melting and metallurgical bonding in the microwave process. Their demonstration provided an approach that resembles a true layer-by-layer AM method.

In a very recent study, Teng et al. used focused microwaves, from a metamaterial near-field electromagnetic structured device, to allow for 3D printing of spatially freely formed objects from metallic nanoparticle inks, even when the nanomaterials were completely encapsulated in a non-absorbing polymeric material [114]. In their approach, a structure was designed that could deliver significant microwave energy focused to a submillimeter spot size. The structure was essentially a split ring resonator with a tapered tip which allowed better impedance matching and an efficiency of about 79%. The technique was used to demonstrate the ability to create low resistivity silver lines (below 8 × 10^−3^ ohm-cm) on PMMA, silicone, and polyethylene substrates. These capabilities allow printed conductors to approach the performance of bulk metal, and enable embedding within polymers or biological substrates, which is a unique advantage of the microwave approach.

Photon-based processes are increasingly being used to chemically transform materials deposited using AM techniques, enabling the creation of metal features from non-metallic precursors. For example, in printed electronics, intense pulsed light (IPL) from xenon lamps has already been used to thermally reduce metal oxide or organometallic inks into metallic conductors within milliseconds. For example, flashes of 0.65 ms can convert printed copper (II) formate ink into a pure copper (Cu^0^) pattern by rapid decomposition and in situ reduction, yielding ~70% of bulk electrical conductivity on plastic films [115]. 

Additionally, while copper has been a popular model system for in situ reduction studies, parallel efforts on silver precursors have achieved even higher conductivities. For example, early work by Tanaka et al. (2006) showed for the first time that tightly focused femtosecond lasers could induce two-photon photoreduction of aqueous gold and silver salts to produce continuous 3D conductive wires of silver and gold with resistivities only a few times higher than bulk [27]. Expanding on this, Saha et al. (2019) demonstrated high-speed two-photon reduction of a silver diamine complex to print freeform silver nanowires with electrical conductivity around 20% of bulk silver [116].

Subsequent advances refined this approach by improving spatial resolution and material quality, but the focus has remained primarily on noble metals. Gold microstructures have been additively fabricated by loading photocurable resins with metal salts and using light energy to simultaneously polymerize an acrylate matrix and photochemically reduce embedded gold(III) salt, producing intricate 3D nanocomposites up to 20 wt% gold in situ [29]; Shuklu et al. embedded gold and silver precursors in polymer matrices to fabricate sub-wavelength metal/polymer composites via two-photon lithography, while Blasco et al. demonstrated the fabrication of conductive gold-containing microstructures through simultaneous photopolymerization and photoreduction during laser writing [28,117].

A 2019 review by Waller et al. emphasizes that photoreduction processes are largely limited to noble metals, whereas non-noble metal printing typically requires hybrid post-processing to achieve fully metallic phases due to unfavorable redox thermodynamics, rapid reoxidation, and sluggish reaction kinetics [118]. A widely used strategy involves printing a polymer scaffold loaded with metal salts, followed by high temperature pyrolysis and reductive annealing to remove organics and convert the precursor into a metallic phase [119]. Such routes are inherently multi-step and fall short of true single step photochemical AM.

Recent work has begun to overcome the “noble metal barrier”, enabling direct or near-direct photochemical printing of non-noble metals under ambient or mild conditions. One example is in the development of new photoresin chemistries that combine photoredox catalysis with advanced excitation schemes. Kühl et al. reported a photosensitive resin incorporating nickel salts, photoredox catalysts, and sensitized triplet-triplet annihilation upconversions to enable direct laser writing of ferromagnetic nickel micro-architectures under ambient conditions without subsequent furnace reduction [120]. In parallel, approaches bypassing polymer matrices all together have emerged with a demonstration of polymer-free nanoscale metal printing by using ultrafast lasers to photolyze metal carbonyl precursors and optically assemble the liberated atoms into dense metallic nanostructures, including molybdenum-based and cobalt-containing alloys in a single step [121]. Along those same lines, Wei et al. leveraged femtosecond laser-induced hot electron processes in plasmonic systems to drive multi-electron reduction and sintering of metal nanoparticles, producing copper and gold microstructures with conductivities approaching bulk powders [122].

#### 3.1.3. Additive Manufacturing of Semiconductors

Traditional solution-processed semiconductor films (e.g., metal oxides or 2D nanomaterials) often require post-processing to achieve adequate electronic performance, which can be incompatible with polymeric substrates. As a result, a major focus for printed semiconductor development is reducing or eliminating these additional steps. Additionally, nanosheet-based thin films printed from solution-processed 2D semiconductor inks have demonstrated significant limitations in achieving electronic properties comparable to their bulk counterparts [123]. However, coupling photo-induced reactions with deposition would enable researchers to directly produce high-quality semiconductors on low-cost, heat sensitive platforms [124]. Truly in situ photo-induced synthesis of semiconductors of multi-phase functional materials is still in its early stages and comprehensive references remain scarce. The following studies, while not fulling integrated, provide valuable insight into how photo-induced mechanisms could eventually be incorporated within AM processes for in situ semiconductor synthesis.

Intense pulsed light (IPL) has been used to simultaneously anneal printed solution-derived indium-gallium-zinc oxide (IGZO) and sinter-printed silver contacts to yield an “all printed” thin-film transistor with performance comparable to conventionally fabricated devices. Using IPL enables spatially selective and extremely fast exposure to achieve oxide lattice condensation and metal conductivity without overheating a flexible substrate [124].

Recently, researchers achieved one-step growth of single-crystal silicon (Si) by laser writing. Typically, attempts to laser deposit crystalline Si from conventional sources (e.g., silicon nanoparticle inks or low-molecular weight silane gas) yield polycrystalline or amorphous Si due to the high decomposition temperatures and rapid cooling, which impede long-range order. Utilizing laser-induced photochemical reactions (nanosecond UV or green laser) and a liquid precursor solution resulted in thermal decomposition of the precursor, and simultaneously induced epitaxial crystallization of silicon onto a seed wafer [125].

Solution-deposited chalcogenide precursors have been converted to functional semiconductor films by scanning lasers as demonstrated through in situ photothermal conversion of metal sulfur inks. These efforts have produced uniform polycrystalline MoS_2_ thin-film electronics (spin coating) [126]. In the realm of optoelectronic semiconductors, recent work has combined direct-write printing with photochemistry to create halide perovskite quantum dot microarrays [127]. For this, an inkjet depositing a lead halide salt resin within a polymer matrix, followed by exposing each wet droplet to UV light, triggered in situ crystallization of the perovskite nanocrystals within the polymer matrix. This produced hemispherical micro-LED color conversion pixels with bright photoluminescence [127].

#### 3.1.4. Limitations of Additive Manufacturing

While additive manufacturing poses as an environmentally friendly, transformative, and efficient method of material synthesis, there are limitations to its expansion. Many materials commonly produced by AM require post-production processing to alleviate distortion, poor mechanical properties, or poor surface quality [128]. Many of today’s AM processes rely on highly specialized powders, wires, or resins that must be meticulously prepared and maintained while requiring extensive post-processing to achieve the desired part quality.

A critical feedstock related bottleneck is the limited range and robustness of materials that can be printed, which significantly limits utilizing AM processes to fabricate parts for demanding environments such as nuclear reactors or deep space. The challenges to overcome within the capabilities of precursor materials are extending the shelf-life and increasing the durability. For instance, ultra-high temperature alloys, specialized ceramics and certain composites with the requisite radiation tolerance or thermal stability are not yet available as off-the-shelf AM powders or filaments. Creating new AM feedstocks is often non-trivial, as custom powder production in small batches can take months, with costs reaching multiple times those of standard alloys, and even then the powder may have suboptimal particle size distribution or flow characteristics for existing machines [129]. Additionally, feedstock materials can degrade over time or under suboptimal storage conditions, which raises concerns about reliability, shelf-life and waste. For example, metal powders gradually oxidize and adsorb moisture, which degrades flowability and can introduce porosity and chemistry shifts in printed parts. Polymer filaments and resins can adsorb water as well or undergo partial curing over time, which can lead to embrittlement or viscosity changes that impact the overall printing process.

These challenges motivate a search for more resilient, flexible feedstock solutions. Beyond the aforementioned limitations, many feedstocks and their associated processes are inherently inefficient, as only a fraction of the raw material ends up in the final powder, and energy-intensive thermal post-treatments are frequently needed to reach full density or desired microstructures.

The solution to extend feedstock materials’ capacity is possibly using photons to induce synthesis of inorganic materials by transforming more stable precursors into high-performance materials during the printing process. By expanding the functional capacity of feedstocks by making them easier to store, handle, and procure, in situ photon-induced synthesis could help additive manufacturing overcome current feedstock constraints.

## 4. Conclusions

### 4.1. Challenges and Opportunities in Photo-Induced Chemistry for AM

Photo-induced chemical processes have great potential to provide new and more efficient pathways for the realization of advanced materials. Additionally, it is poised to redefine 3D printing from a geometry-centric process into a unified platform for materials synthesis. The combination of advanced manufacturing technology with laser-light-induced oxidation–reduction and combustion reactions offers significant promise toward more efficient, pointed, and purposeful production of catalysts, polymers, metal films, ceramics, and semiconductors. However, some significant challenges must still be overcome for full realization of photo-induced processing in these areas.

Applying photochemistry with AM in the industry may be limited by the difficulty of scaling up reactions from batch to large-scale production, which requires specialized implementation designs, strong light sources and design considerations to ensure uniform light penetration. Short-wavelength light may be required for some applications and has short penetration depths in some media, and may lead to complications due to side reactions. The efficiency and stability of the process may also be difficult to measure and may depend on the amount of material involved in the AM processing. Photocatalytic processes often deactivate over time and achieving consistent results for light-assisted reactions may be challenging. Also, materials may be expensive, and upfront considerations of ease of recovery of expensive components and minimization of waste will likely prove important considerations for the future. 

### 4.2. Summary

In summary, photo-induced reactions utilize light sources like lasers to synthesize materials. Photo-induced reactions overcome the use of typical wet chemistry methods, making it a more efficient and environmentally friendly method of synthesis. Reactions reviewed in this paper include oxidation–reduction, combustion, decomposition, and polymerization reactions. Materials synthesized range from metals to ceramics. Such materials are often used in additive manufacturing applications. In such applications, limitations within feedstock and product quality call for a novel approach to synthesis. With increased use of photo-induced reactions as a methodology that can contribute alternative synthetic ideas, the problem of feedstock limitations may be partially solved through the world of lasers and photons.

## Figures and Tables

**Table 1:** Photo-induced mechanisms [1].

Mechanism	Laser Regime & Energy Delivery	Dominant Energy Pathway	Representative Outcomes
**Photothermal** [1,2]	Longer pulses (nanoseconds to continuous wave) Slower repetition or overlapping pulses for heat accumulation	Heat (phonons): Absorbed light rapidly converts to lattice and substrate heat, raising local temperature.	Melting & resolidification; sintering/fusing of particles Phase transformations (e.g., crystallization, alloying) Localized thermal decomposition or evaporation.
**Photochemical** [3,4]	Broad range of pulse lengths (femtoseconds to continuous) as long as photon energy (or multiphoton equivalent) exceeds bond energies. Often UV or spectral absorption match needed. Typically low fluence just above reaction threshold.	Electronic excitation: Photons break chemical bonds or drive reactions directly, with minimal bulk heating, e.g., multiphoton absorption, resonant excitation of specific bonds, generation of excited-state species/radicals.	Bond cleavage and ablation of organics (photolysis) Laser-induced reduction/oxidation of metals and compounds at low T polymerization or cross-linking (photo-curing of resins) removal of surface ligands (photo-cleavage) leading to chemical patterning.
**Plasma-mediated** [5,6]	Ultrashort pulses (fs–ps) or high-peak-power Q-switched ns High intensity (>10^12^ W/cm^2^) for optical breakdown; Tight focusing (small spot) often required. Frequently under vacuum or controlled gas for plasma consistency.	Ionization & plasma formation: Intense field creates free electrons and an ionized plasma, with energy transfer via kinetic ions, shock waves, and bright plasma radiation. Minimal initial thermal diffusion (energy deposited faster than heat can spread).	Ablation & exfoliation (material removed by Coulomb explosion and shock pressure) Plasma-assisted surface modification (creates defects, surface activation sites, or phase densification) Ultrafast quenching leading to amorphous or nanocrystalline phases Synthesis of nanoparticles or new compounds in the plasma plume.

**Table 2:** Laser summary.

Laser	Characteristics	Wavelengths	Uses
**Continuous Wave (CW)**	**Continuously Pumped and Continuously Emits Light**		
Water-Cooled Ion (Ar)	Water-cooled tube produces argon plasma	Ultraviolet—green many lines (351.1–528.7 nm)	Pumping, Raman spectroscopy, and lithography.
Water-Cooled Ion (Kr)	Water-cooled tube produces krypton plasma	Violet—red many lines (415.1–676.4 nm)	Flow cytometry, surgery, and confocal microscopy.
CO_2_	Highest power CW laser	Infrared lines (9–11 µm)	Industrial cutting, welding, additive manufacturing, and dermatology.
**Diode**	**Semiconductor Device that Emits Light with the Same Phase and Wavelength**		
Diode-Pumped Solid-State	Solid-state laser pumped with diode	Varies	Additive manufacturing, stereolithography, and micromachining.
**Pulsed**	**Stores Energy and Emits Light in High Energy Pulses**		
Flash-Pumped Dye	Uses liquid gain medium	Visible range	Skin condition treatment.
Solid-State	Uses solid gain medium	0.7 to 2.1 μm	Cutting, welding, surgery, and spectroscopy.
Fiber	Uses optic fiber gain medium	220–780 nm	Materials processing, medicine, and microscopy.
Excimer	Pulsed gas that emits UV	193 to 350 nm	Semiconductor production, surgery, and skin treatment.

**Table 3:** Merits and demerits of photo-induced reaction system [54,55].

Merits	Demerits
Not typically reliant on harsh solvent Often carried out at room temperature Characteristically less waste produced More environmentally friendly Focused EM allows for spatial control Temporal control enables rapid, clean process Pinpoint focus drives microscale reactions Combine w/AM to Fabricate Complex parts Allows for non-equilibrium synthesis Tunable energy input allows scalability	Excitation may lead to unwanted reaction pathways Photochemical side reactions could limit efficiency Photo-decomposition of catalyst may occur Photo-induced mechanisms are often complicated Non-uniform light can cause suboptimal results Incorrect frequency range leads to large inefficiency Laser and EM equipment is often costly Specialized safety precautions are often required

**Table 4:** Photo-induced reactions summary table.

Reaction Type	Precursor	Light Source	Material	Advantages	Relevance to AM	Ref
Laser-induced redox reaction (CuO → Cu)	CuO nanoparticle ink	Yb-doped fiber laser 1070 nm	Cu metal film	Rapid local reduction; patternable	In situ metallization	[67]
Photoreduction from electrolyte	CuSO_4_, CuCl_2_	Ar^+^ laser multi-line	Cu metal	Low-temp, autocatalytic	Localized metal deposition	[68]
Facet-selective Cu_2_O transformation	Cu_2_O microcrys-tals	Broadband lamp + bias	Hollow Cu nanoshells	Facet-specific morphology control	Microstructure tuning	[71]
Laser-induced metal deposition on graphene	Metal ions (Au, Ag, Pd, Pb, Pt)	532 nm laser	Metal nanopattern	Nanoscale patterning	Printed electronics	[72]
Laser-induced GO reduction	Graphite oxide	808 nm CW laser	Graphene	Chemical-free; rapid	On-substrate graphene formation	[73]
UV pulsed GO reduction	Graphene oxide powders	UV pulsed laser	Reduced GO	Controlled oxygen removal	Tunable conductivity	[74]
CO_2_ line-beam GO reduction	GO films	CO_2_ laser at 10.6 µm	Reduced GO films	High throughput	Large-area film conversion	[75]
Femtosecond N-doping of GO	GO + NH_3_	800 nm fs laser	N-doped graphene	Precise, localized doping	Voxel-level electronic tuning	[76]
UV PDC polymerization	Borazine pre-ceramic monomer	UV lamp	Si/B/C/N ceramic	Fast curing	Supports ceramic AM	[83]
Photo-reversible cross-linking	PVA–coumarin resin	UV light at 254/354 nm	Reversible polymer	Better adhesion; re-workability	SLA/DLP enhancement	[85]
Light-assisted MnOx synthesis	Mn precursor solutions	405 nm LED	MnOx films	Tuned morphology	Functional coatings	[86]
UV-triggered ZnO NP assembly	ZnO + citrate	Low-intensity UV	ZnO stacked layers	Charge-control assembly	Semiconductor patterning	[87]
Laser-induced Cu oxidation	Cu target + oxidizers	Excimer laser 193 & 308 nm	CuO	Tunable oxidation	Metal oxide feedstock	[66]
LIB halide reactions	BCl_3_, BF_3_ + H_2_	Nd:YAG laser 1064 nm	B, B_4_C	Direct ceramic formation	Hard coatings for AM	[77]
Microwave SHS (TiC/Al_2_O_3_)	TiO_2_ + C + Al	2.45 GHz microwave	TiC + Al_2_O_3_	Volumetric heating	Reactive ceramic printing	[80]
CO_2_ photo-decomposed	C_2_H_6_/SF_6_; SiH_4_	CO_2_ laser at 10 µm	C & Si clusters	Ultrapure nanoparticle synthesis	AM ink feedstock	[82]
Two-Photon Photoreduction (2PP)	Ag^+^, Au^3+^ precursors	780 nm fs laser	Ag/Au microstructures	Sub-micron precision	In situ metal printing	[27]
Dual photopolymerized/photoreduced	Au(III) in resin	800 nm fs laser	Au–polymer composites	One-step hybrid synthesis	Metal-polymer microfeatures	[29]

## Data Availability

No new data were created or analyzed in this study. Data sharing is not applicable to this article.

## References

[1] Bäuerle D. (1996). Laser Processing and Chemistry.

[2] Stuart B.C., Feit M.D., Rubenchik A.M., Shore B.W., Perry M.D. (1995). Laser-Induced Damage in Dielectrics with Nanosecond to Subpicosecond Pulses. Phys. Rev. Lett..

[3] Srinivasan R., Braren B. (1989). Ultraviolet Laser Ablation of Organic Polymers. Chem. Rev..

[4] Fatkullin M., Cheshev D., Averkiev A., Gorbunova A., Murastov G., Liu J., Postnikov P., Cheng C., Rodriguez R.D., Sheremet E. (2024). Photochemistry Dominates over Photothermal Effects in the Laser-Induced Reduction of Graphene Oxide by Visible Light. Nat. Commun..

[5] Pronko P.P., VanRompay P.A., Horvath C., Loesel F., Juhasz T., Liu X., Mourou G. (1998). Avalanche Ionization and Dielectric Breakdown in Silicon with Ultrafast Laser Pulses. Phys. Rev. B.

[6] Sugioka K., Cheng Y. (2014). Ultrafast Lasers—Reliable Tools for Advanced Materials Processing. Light Sci. Appl..

[7] Bloembergen N., Hall P. (1999). From Nanosecond to Femtosecond Science. Rev. Mod. Phys..

[8] (2020). Sixty Years of Lasers. Nat. Rev. Phys..

[9] Jauregui C., Limpert J., Tünnermann A. (2013). High-Power Fibre Lasers. Nat. Photonics.

[10] Zhou S., Cao J., Chen M., Wang Z., Si L., Chen J. (2024). Review of High-Power Continuous Wave Yb-Doped Fiber Lasers near 980 Nm. Photonics.

[11] Fan T.Y., Byer R.L. (1988). Diode Laser-Pumped Solid-State Lasers. IEEE J. Quantum Electron..

[12] Wang G., Song J., Chen Y., Ren S., Ma P., Liu W., Yao T., Zhou P. (2022). Six Kilowatt Record All-Fiberized and Narrow-Linewidth Fiber Amplifier with near-Diffraction-Limited Beam Quality. High Power Laser Sci. Eng..

[13] Malinauskas M., Žukauskas A., Hasegawa S., Hayasaki Y., Mizeikis V., Buividas R., Juodkazis S. (2016). Ultrafast Laser Processing of Materials: From Science to Industry. Light Sci. Appl..

[14] Gautam G.D., Pandey A.K. (2018). Pulsed Nd:YAG Laser Beam Drilling: A Review. Opt. Laser Technol..

[15] Tsai M.-J., Wu L.-F. (2022). Multi-Objective Optimization of Nd:YAG Laser Drilling of Optical-Grade Acrylic Plate Using Taguchi-Based Grey Relational Analysis. Materials.

[16] Qin Y., Qi Q., Shi P., Scott P.J., Jiang X. (2021). Automatic Determination of Part Build Orientation for Laser Powder Bed Fusion. Virtual Phys. Prototyp..

[17] King W.E., Anderson A.T., Ferencz R.M., Hodge N.E., Kamath C., Khairallah S.A., Rubenchik A.M. (2015). Laser Powder Bed Fusion Additive Manufacturing of Metals; Physics, Computational, and Materials Challenges. Appl. Phys. Rev..

[18] Ye L., Xue H., Li Z., Zhou Y., Chen G., Xu F., Melentiev R., Newman S., Yu N. (2025). Review of Online Quality Control for Laser Directed Energy Deposition (LDED) Additive Manufacturing. Int. J. Extrem. Manuf..

[19] Huang J., Qin Q., Wang J. (2020). A Review of Stereolithography: Processes and Systems. Processes.

[20] Schwartz J.J. (2022). Additive Manufacturing: Frameworks for Chemical Understanding and Advancement in Vat Photopolymerization. MRS Bull..

[21] O’Halloran S., Pandit A., Heise A., Kellett A. (2023). Two-Photon Polymerization: Fundamentals, Materials, and Chemical Modification Strategies. Adv. Sci..

[22] Göppert-Mayer M. (1931). Über Elementarakte mit zwei Quantensprüngen. Ann. Phys..

[23] Harinarayana V., Shin Y.C. (2021). Two-Photon Lithography for Three-Dimensional Fabrication in Micro/Nanoscale Regime: A Comprehensive Review. Opt. Laser Technol..

[24] Tanaka T., Sun H.-B., Kawata S. (2002). Rapid Sub-Diffraction-Limit Laser micro/nanoprocessing in a Threshold Material System. Appl. Phys. Lett..

[25] Sun H.-B., Kawata S. (2006). Two-Photon Photopolymerization and 3D Lithographic Microfabrication. NMR 3D Analysis Photopolymerization.

[26] Farsari M., Chichkov B.N. (2009). Two-Photon Fabrication. Nat. Photonics.

[27] Tanaka T., Ishikawa A., Kawata S. (2006). Two-Photon-Induced Reduction of Metal Ions for Fabricating Three-Dimensional Electrically Conductive Metallic Microstructure. Appl. Phys. Lett..

[28] Shukla S., Furlani E.P., Vidal X., Swihart M.T., Prasad P.N. (2010). Two-Photon Lithography of Sub-Wavelength Metallic Structures in a Polymer Matrix. Adv. Mater..

[29] Hu Q., Sun X.-Z., Parmenter C.D.J., Fay M.W., Smith E.F., Rance G.A., He Y., Zhang F., Liu Y., Irvine D. (2017). Additive Manufacture of Complex 3D Au-Containing Nanocomposites by Simultaneous Two-Photon Polymerisation and Photoreduction. Sci. Rep..

[30] Desponds A., Banyasz A., Montagnac G., Andraud C., Baldeck P., Parola S. (2020). Microfabrication by Two-Photon Lithography, and Characterization, of SiO2/TiO2 Based Hybrid and Ceramic Microstructures. J. Sol-Gel Sci. Technol..

[31] Balčas G., Malinauskas M., Farsari M., Juodkazis S. (2023). Fabrication of Glass-Ceramic 3D Micro-Optics by Combining Laser Lithography and Calcination. Adv. Funct. Mater..

[32] Chen Z., Li Z., Li J., Liu C., Lao C., Fu Y., Liu C., Li Y., Wang P., He Y. (2019). 3D Printing of Ceramics: A Review. J. Eur. Ceram. Soc..

[33] Pham T.A., Kim D.-P., Lim T.-W., Park S.-H., Yang D.-Y., Lee K.-S. (2006). Three-Dimensional SiCN Ceramic Microstructures via Nano-Stereolithography of Inorganic Polymer Photoresists. Adv. Funct. Mater..

[34] Kotz F., Arnold K., Bauer W., Schild D., Keller N., Sachsenheimer K., Nargang T.M., Richter C., Helmer D., Rapp B.E. (2017). Three-Dimensional Printing of Transparent Fused Silica Glass. Nature.

[35] Luo J., Park J.A., Baek S., Kim S., Jeong U., Jung S. (2026). Functional Nano-to-Microstructures by Jet Printing and Direct Ink Writing. Chem. Rev..

[36] Chatterjee C., Lupoi R., Gibbons M.J. (2026). The Evolution of Aerosol Jet Printing, A Review: Enhancing Material Versatility and Improvements for Next-Generation Applications. Adv. Mater. Technol..

[37] Fujimoto K.T., Hone L.A., Manning K.D., Seifert R.D., Davis K.L., Milloway J.N., Skifton R.S., Wu Y., Wilding M., Estrada D. (2021). Additive Manufacturing of Miniaturized Peak Temperature Monitors for In-Pile Applications. Sensors.

[38] Fujimoto K.T., Watkins J.K., Phero T., Litteken D., Tsai K., Bingham T., Ranganatha K.L., Johnson B.C., Deng Z., Jaques B. (2020). Aerosol Jet Printed Capacitive Strain Gauge for Soft Structural Materials. npj Flex. Electron..

[39] Patel A., Taufik M. (2024). Extrusion-Based Technology in Additive Manufacturing: A Comprehensive Review. Arab. J. Sci. Eng..

[40] Clarkson C.M., Wyckoff C., Parvulescu M.J.S., Rueschhoff L.M., Dickerson M.B. (2022). UV-Assisted Direct Ink Writing of Si3N4/SiC Preceramic Polymer Suspensions. J. Eur. Ceram. Soc..

[41] Pandhi T., Cornwell C., Fujimoto K., Barnes P., Cox J., Xiong H., Davis P.H., Subbaraman H., Koehne J.E., Estrada D. (2020). Fully Inkjet-Printed Multilayered Graphene-Based Flexible Electrodes for Repeatable Electrochemical Response. RSC Adv..

[42] Gupta A.A., Arunachalam S., Cloutier S.G., Izquierdo R. (2018). Fully Aerosol-Jet Printed, High-Performance Nanoporous ZnO Ultraviolet Photodetectors. ACS Photonics.

[43] Ouyang J., Cormier D., Williams S.A., Borkholder D.A. (2016). Photonic Sintering of Aerosol Jet Printed Lead Zirconate Titanate (PZT) Thick Films. J. Am. Ceram. Soc..

[44] Nelson M.L., Burgoyne H., Kouchi F.R., White F., Rektor A., Fujimoto K., Sawyer M., Tibbits A., Toodeshki E.H., Semodji A.M. (2026). Graphene and Ti_3_C_2_T_*x*_ MXene Nanomaterial-Infused Bioinks for Mechanical and Electrical Improvement of 3D Bioprinted Scaffolds. ACS Appl. Bio Mater..

[45] Kouchi F.R., Varghese T.V., Burgoyne H., Mansoor N.E., Seol M., McKibben N., Nirantar S., Chinnathambi K., Eixenberger J., Maryon O. (2025). StableTi_3_C_2_T_*x*_ MXene Ink Formulation and High-Resolution Aerosol Jet Printing for High-Performance MXene Supercapacitors. Small Methods.

[46] Yi H., Liu Y., Cao H., Luo J., Dong X., An J., Chua C.K. (2025). Material and Process Integrated Innovations in Aerosol Jet Printing: A Review. Mater. Today.

[47] Nabi S., Isaev A., Chiolerio A. (2024). Inkjet Printing of Functional Materials for Low-Temperature Electronics: A Review of Materials and Strategies. ACS Appl. Electron. Mater..

[48] Rajeshirke M., Fidan I., Naikwadi V., Alkunte S., Gupta A., Mohammadizadeh M. (2025). Material Extrusion–Based Multi-Material 3D Printing: A Holistic Review of Recent Advances. Int. J. Adv. Manuf. Technol..

[49] Gianvittorio S., Tonelli D., Lesch A. (2023). Print-Light-Synthesis for Single-Step Metal Nanoparticle Synthesis and Patterned Electrode Production. Nanomaterials.

[50] Zhang Y., Liu C., Whalley D. Direct-Write Techniques for Maskless Production of Microelectronics: A Review of Current State-of-the-Art Technologies. Proceedings of the 2009 International Conference on Electronic Packaging Technology & High Density Packaging.

[51] Ermak O., Zenou M., Toker G.B., Ankri J., Shacham-Diamand Y., Kotler Z. (2016). Rapid Laser Sintering of Metal Nano-Particles Inks. Nanotechnology.

[52] Chen M., Liu X., Wang Y., Hu Z., Hua L., Hu Z., Qin X., Huang Y. (2026). Challenges, Solutions, and Future Perspectives in Metal Additive Manufacturing: A Review. J. Mater. Eng. Perform..

[53] Calignano F. (2018). Investigation of the Accuracy and Roughness in the Laser Powder Bed Fusion Process. Virtual Phys. Prototyp..

[54] Bongiovanni R., Vacche S.D., Vitale A. (2021). Photoinduced Processes as a Way to Sustainable Polymers and Innovation in Polymeric Materials. Polymers.

[55] Hennig H., Billing R. (1993). Advantages and Disadvantages of Photocatalysis Induced by Light-Sensitive Coordination Compounds. Coord. Chem. Rev..

[56] Smith D., McCartney M., Bursill L. (1987). The electron-beam-induced reduction of transition metal oxide surfaces to metallic lower oxides. Ultramicroscopy.

[57] Al-Otaify A. (2025). Review on Continuous-Wave Laser Ablation for Nanoparticle Production in Liquids. Plasmonics.

[58] Solati E., Mashayekh M., Dorranian D. (2013). Effects of Laser Pulse Wavelength and Laser Fluence on the Characteristics of Silver Nanoparticle Generated by Laser Ablation. Appl. Phys. A.

[59] Rahman A., Guisbiers G. (2024). Synthesis of Nickel-Based Nanoparticles by Pulsed Laser Ablation in Liquids: Correlations between Laser Beam Power, Size Distribution and Cavitation Bubble Lifetime. Metals.

[60] Desarkar H.S., Kumbhakar P., Mitra A.K. (2012). Effect of Ablation Time and Laser Fluence on the Optical Properties of Copper Nano Colloids Prepared by Laser Ablation Technique. Appl. Nanosci..

[61] Elsayed K.A., Imam H., Ahmed M.A., Ramadan R. (2013). Effect of Focusing Conditions and Laser Parameters on the Fabrication of Gold Nanoparticles via Laser Ablation in Liquid. Opt. Laser Technol..

[62] Waag F., Streubel R., Gökce B., Barcikowski S. (2021). Synthesis of Gold, Platinum, and Gold-Platinum Alloy Nanoparticle Colloids with High-Power Megahertz-Repetition-Rate Lasers: The Importance of the Beam Guidance Method. Appl. Nanosci..

[63] Shaheen M.E., Abdelwahab A.Y.E. (2025). Laser Ablation in Liquids: A Versatile Technique for Nanoparticle Generation. Opt. Laser Technol..

[64] Abdalameer N.K., Majeed N.F., Buraihi A.K., Ali S.H. (2026). Optical Nanoparticle Synthesis: A Comprehensive Laser Ablation Review. J. Opt..

[65] He Z., Lei L., Lin S., Tian S., Tian W., Yu Z., Li F. (2024). Metal Material Processing Using Femtosecond Lasers: Theories, Principles, and Applications. Materials.

[66] Otis C.E., Gupta A., Braren B. (1993). Gas-Phase Oxidation of Copper during Laser Ablation of YBa2Cu307-6 in Different Oxidizing Ambients. Appl. Phys. Lett..

[67] Kang B., Han S., Kim J., Ko S., Yang M. (2011). One-Step Fabrication of Copper Electrode by Laser-Induced Direct Local Reduction and Agglomeration of Copper Oxide Nanoparticle. J. Phys. Chem. C.

[68] Kim D., Choi C. (2013). Laser-Induced Metal Reduction from Liquid Electrolyte Precursor. J. Nanosci. Nanotechnol..

[69] Langille M.R., Personick M.L., Mirkin C.A. (2013). Plasmon-Mediated Syntheses of Metallic Nanostructures. Angew. Chem. Int. Ed..

[70] Tan C., Qin C., Sadtler B. (2017). Light-Directed Growth of Metal and Semiconductor Nanostructures. J. Mater. Chem. C.

[71] Qin C., Campbell B.M., Shen M., Zhao T., Sadtler B. (2019). Light-Driven, Facet-Selective Transformation of Cuprous Oxide Microcrystals to Hollow Copper Nanoshells. Chem. Mater..

[72] Xia K., Chiang W.-Y., Lockhart De La Rosa C.J., Fujita Y., Toyouchi S., Yuan H., Su J., Masuhara H., De Gendt S., De Feyter S. (2020). Photo-Induced Electrodeposition of Metallic Nanostructures on Graphene. Nanoscale.

[73] Wang D., Min Y., Yu Y., Peng B. (2014). Laser Induced Self-Propagating Reduction and Exfoliation of Graphite Oxide as an Electrode Material for Supercapacitors. Electrochim. Acta.

[74] Yang C.-R., Tseng S.-F., Chen Y.-T. (2018). Laser-Induced Reduction of Graphene Oxide Powders by High Pulsed Ultraviolet Laser Irradiations. Appl. Surf. Sci..

[75] Tran T.X., Choi H., Che C.H., Sul J.H., Kim I.G., Lee S.-M., Kim J.-H., In J.B. (2018). Laser-Induced Reduction of Graphene Oxide by Intensity-Modulated Line Beam for Supercapacitor Applications. ACS Appl. Mater. Interfaces.

[76] Guo L., Zhang Y., Han D., Jiang H., Wang D., Li X., Xia H., Feng J., Chen Q., Sun H. (2014). Laser-Mediated Programmable N Doping and Simultaneous Reduction of Graphene Oxides. Adv. Opt. Mater..

[77] Gornushkin I.B., Sennikov P.G., Kornev R.A., Ermakov A.A., Shkrunin V.E. (2020). Laser Induced Dielectric Breakdown for Chemical Vapor Deposition by Hydrogen Reduction of Volatile Boron Halides BCl3 and BF3. Plasma Chem. Plasma Process..

[78] A. Kornev R., Sennikov P.G., Gornushkin I.B., A. Ermakov A., Shkrunin V.E., Polykov V.S., Kornev A.R., Kornev K.D. (2022). Laser Induced Dielectric Breakdown as a Novel Method for the Synthesis of Molybdenum Boride. Plasma Chem. Plasma Process..

[79] Alexandrescu R., Borsella E., Botti S., Cesile M.P., Martelli S., Giorgi R., Turtù S., Zappa G. (1997). Synthesis of Aluminum Oxide-Based Ceramics by Laser Photoinduced Reactions from Gaseous Precursors. J. Mater. Res..

[80] Yiin T., Barmatz M., Feng H., Moore J. (1995). Microwave Induced Combustion Synthesis of Ceramic and Ceramic-Metal Composites.

[81] Kim S.-B. (2002). Synthesis of Al_2_O_3_/SiC Composites by the SHS Microwave Heating Process. Met. Mater. Int..

[82] Ehbrecht M., Ferkel H., Smirnov V.V., Stelmakh O., Zhang W., Huisken F. (1996). Laser-driven synthesis of carbon and silicon clusters from gas-phase reactants. Surf. Rev. Lett..

[83] Wei D., Chen L., Xu T., He W., Wang Y. (2016). Synthesis and Characterization of a Novel Borazine-Type UV Photo-Induced Polymerization of Ceramic Precursors. Molecules.

[84] Calderón-Villajos R., López A.J., Peponi L., Manzano-Santamaría J., Ureña A. (2019). 3D-Printed Self-Healing Composite Polymer Reinforced with Carbon Nanotubes. Mater. Lett..

[85] Xue H., Li X., Xia J., Lin Q. (2020). A Photo-Reversible Crosslinking Resin for Additive Manufacturing: Reversibility and Performance. RSC Adv..

[86] Qin C. (2021). Light-Directed Growth of Semiconductor Nanomaterials by Photoelectrodeposition. Ph.D. Thesis.

[87] He X., Gu H., Ma Y., Cai Y., Jiang H., Zhang Y., Xie H., Yang M., Fan X., Guo L. (2024). Light Patterning Semiconductor Nanoparticles by Modulating Surface Charges. Nat. Commun..

[88] Zhou D., Gao Y., Liu H., Zhang G., Li L. (2019). Light-Induced Patterned Self-Assembly Behavior of Isotropic Semiconductor Nanomotors. Chem. Asian J..

[89] Ahmed S.E. (2022). Light Induced Effects in Oxide Semiconductors.

[90] Sakaguchi Y., Tenne D.A., Mitkova M. (2009). Oxygen-assisted Photoinduced Structural Transformation in Amorphous Ge–S Films. Phys. Status Solidi B.

[91] Mak K.F., Lee C., Hone J., Shan J., Heinz T.F. (2010). Atomically Thin MoS 2: A New Direct-Gap Semiconductor. Phys. Rev. Lett..

[92] Klein J., Wierzbowski J., Regler A., Becker J., Heimbach F., Müller K., Kaniber M., Finley J.J. (2016). Stark Effect Spectroscopy of Mono- and Few-Layer MoS_2_. Nano Lett..

[93] Akinwande D., Huyghebaert C., Wang C.-H., Serna M.I., Goossens S., Li L.-J., Wong H.-S.P., Koppens F.H.L. (2019). Graphene and Two-Dimensional Materials for Silicon Technology. Nature.

[94] Chaves A., Azadani J.G., Alsalman H., Da Costa D.R., Frisenda R., Chaves A.J., Song S.H., Kim Y.D., He D., Zhou J. (2020). Bandgap Engineering of Two-Dimensional Semiconductor Materials. npj 2D Mater. Appl..

[95] Hiraoka T., Nestler S., Zhang W., Rossel S., Hafez H.A., Fabretti S., Schlörb H., Thomas A., Turchinovich D. (2025). Terahertz Field Effect in a Two-Dimensional Semiconductor. Nat. Commun..

[96] Alberi K., Scarpulla M.A. (2018). Photoassisted Physical Vapor Epitaxial Growth of Semiconductors: A Review of Light-Induced Modifications to Growth Processes. J. Phys. Appl. Phys..

[97] Bonfield H.E., Knauber T., Lévesque F., Moschetta E.G., Susanne F., Edwards L.J. (2020). Photons as a 21st Century Reagent. Nat. Commun..

[98] Yee D.W., Greer J.R. (2021). Three-dimensional Chemical Reactors: *In Situ* Materials Synthesis to Advance Vat Photopolymerization. Polym. Int..

[99] Zeng Y., Wang J., Liu X., Xue Y., Tang L., Tong Y., Jiang F. (2024). Laser Additive Manufacturing of Ceramic Reinforced Titanium Matrix Composites: A Review of Microstructure, Properties, Auxiliary Processes, and Simulations. Compos. Part Appl. Sci. Manuf..

[100] Montón Zarazaga A., Abdelmoula M., Küçüktürk G., Maury F., Ferrato M., Grossin D. (2022). Process Parameters Investigation for Direct Powder Bed Selective Laser Processing of Silicon Carbide Parts. Prog. Addit. Manuf..

[101] Montón A., Maury F., Chevallier G., Estournès C., Ferrato M., Grossin D. (2025). Core–Shell Powder Strategy for Additive Manufacturing of Ceramics: Application to Direct Powder Bed Selective Laser Processing of Silicon Carbide. J. Aust. Ceram. Soc..

[102] Abdelmoula M., Küçüktürk G., Juste E., Petit F. (2022). Powder Bed Selective Laser Processing of Alumina: Scanning Strategies Investigation. Appl. Sci..

[103] Liu Q., Danlos Y., Song B., Zhang B., Yin Y., Liao H. (2015). Effect of high-temperature preheating on the selective laser melting of yttria-stabilized zirconia ceramic. J. Mater. Process. Technol..

[104] Herzog D., Seyda V., Wycisk E., Emmelmann C. (2016). Additive Manufacturing of Metals. Acta Mater..

[105] Clark E. (1994). Microwave Processing of Materials.

[106] Brown M.S., Arnold C.B., Sugioka K., Meunier M., Piqué A. (2010). Fundamentals of Laser-Material Interaction and Application to Multiscale Surface Modification. Laser Precision Microfabrication.

[107] Roy R., Agrawal D., Cheng J., Gedevanishvili S. (1999). Full Sintering of Powdered-Metal Bodies in a Microwave Field. Nature.

[108] Oghbaei M., Mirzaee O. (2010). Microwave versus Conventional Sintering: A Review of Fundamentals, Advantages and Applications. J. Alloys Compd..

[109] Shoshani Y., Weinstein T., Barkay Z., Jerby E. (2023). Sequential Solidification of Metal Powder by a Scanning Microwave Applicator. Materials.

[110] Srinath M.S., Sharma A.K., Kumar P. (2011). A New Approach to Joining of Bulk Copper Using Microwave Energy. Mater. Des..

[111] Singh S., Gupta D., Jain V. (2016). Recent Applications of Microwaves in Materials Joining and Surface Coatings. Proc. Inst. Mech. Eng. Part B J. Eng. Manuf..

[112] Mishra R.R., Sharma A.K. (2016). A Review of Research Trends in Microwave Processing of Metal-Based Materials and Opportunities in Microwave Metal Casting. Crit. Rev. Solid State Mater. Sci..

[113] Shelef A., Jerby E. (2020). Incremental Solidification (toward 3D-Printing) of Metal Powders by Transistor-Based Microwave Applicator. Mater. Des..

[114] Teng J., Hales S.H., Yang X., Anklam J., Lee S., Liu Y., Sahu D.P., Li L., Latham C., Tian X. (2026). Three-Dimensional Printing of Nanomaterials-Based Electronics with a Metamaterial-Inspired near-Field Electromagnetic Structure. Sci. Adv..

[115] Rosen Y.S., Yakushenko A., Offenhäusser A., Magdassi S. (2017). Self-Reducing Copper Precursor Inks and Photonic Additive Yield Conductive Patterns under Intense Pulsed Light. ACS Omega.

[116] Saha S.K., Au B., Oakdale J.S. (2019). High-Speed Direct Laser Writing of Silver Nanostructures via Two-Photon Reduction. Adv. Eng. Mater..

[117] Blasco E., Müller J., Müller P., Trouillet V., Schön M., Scherer T., Barner-Kowollik C., Wegener M. (2016). Fabrication of Conductive 3D Gold-Containing Microstructures via Direct Laser Writing. Adv. Mater..

[118] Waller E.H., Dix S., Gutsche J., Widera A., Von Freymann G. (2019). Functional Metallic Microcomponents via Liquid-Phase Multiphoton Direct Laser Writing: A Review. Micromachines.

[119] Vyatskikh A., Delalande S., Kudo A., Zhang X., Portela C.M., Greer J.R. (2018). Additive Manufacturing of 3D Nano-Architected Metals. Nat. Commun..

[120] Kühl K.E.J., Rediger K., Khera N., Spindler E., Niedner-Schatteburg G., Neu E., Weiler M., von Freymann G. (2026). Direct Laser Writing of Ferromagnetic Nickel Utilizing the Principle of Sensitized Triplet-Triplet Annihilation Upconversion. arXiv.

[121] Wang Y., Yi C., Tian W., Liu F., Cheng G.J. (2024). Free-Space Direct Nanoscale 3D Printing of Metals and Alloys Enabled by Two-Photon Decomposition and Ultrafast Optical Trapping. Nat. Mater..

[122] Wei K., Chang K., Wang X., Lan S., Hipwell M.C., Pan H. (2026). 3D Nanoprinting of Metals by Spatiotemporally Confined Hot Electrons via Multiple-Electron Excitations in Nanocrystals. Nat. Commun..

[123] Conti S., Calabrese G., Parvez K., Pimpolari L., Pieri F., Iannaccone G., Casiraghi C., Fiori G. (2023). Printed Transistors Made of 2D Material-Based Inks. Nat. Rev. Mater..

[124] Moon C.-J., Park J.-W., Jang Y.-R., Kim H.-S. (2024). Intense Pulsed Light Annealing of Solution-Based Indium–Gallium–Zinc–Oxide Semiconductors with Printed Ag Source and Drain Electrodes for Bottom Gate Thin Film Transistors. Sci. Rep..

[125] Yang X.J., Chen P., Liu Z.K. (2025). Laser Direct Writing and Doping of Single-Crystal Silicon Using Liquid Silanes. J. Phys. Conf. Ser..

[126] Xu M., Gao J., Song J., Wang H., Zheng L., Wei Y., He Y., Wang X., Huang W. (2021). Programmable Patterned MoS2 Film by Direct Laser Writing for Health-Related Signals Monitoring. iScience.

[127] Liu X., Li J., Zhang P., Lu W., Yang G., Zhong H., Zhao Y. (2022). Perovskite Quantum Dot Microarrays: In Situ Fabrication via Direct Print Photopolymerization. Nano Res..

[128] Marya M., Singh V., Marya S., Hascoet J.Y. (2015). Microstructural Development and Technical Challenges in Laser Additive Manufacturing: Case Study with a 316L Industrial Part. Metall. Mater. Trans. B.

[129] Chen H., Kosiba K., Suryanarayana C., Lu T., Liu Y., Wang Y., Prashanth K.G. (2023). Feedstock Preparation, Microstructures and Mechanical Properties for Laser-Based Additive Manufacturing of Steel Matrix Composites. Int. Mater. Rev..

